# Integration of Transcriptome and Whole Genomic Resequencing Data to Identify Key Genes Affecting Swine Fat Deposition

**DOI:** 10.1371/journal.pone.0122396

**Published:** 2015-04-07

**Authors:** Kai Xing, Feng Zhu, Liwei Zhai, Huijie Liu, Yuan Wang, Zhijun Wang, Shaokang Chen, Zhuocheng Hou, Chuduan Wang

**Affiliations:** 1 National Engineering Laboratory for Animal Breeding and MOA Key Laboratory of Animal Genetics and Breeding, Department of Animal Genetics and Breeding, China Agricultural University, Beijing, 100193, China; 2 Tianjin Ninghe primary pig breeding farm, Ninghe, 301500, Tianjin, China; 3 Animal husbandry and veterinary station of Beijing, Beijing, 100125, Beijing, China; Wageningen UR Livestock Research, NETHERLANDS

## Abstract

Fat deposition is highly correlated with the growth, meat quality, reproductive performance and immunity of pigs. Fatty acid synthesis takes place mainly in the adipose tissue of pigs; therefore, in this study, a high-throughput massively parallel sequencing approach was used to generate adipose tissue transcriptomes from two groups of Songliao black pigs that had opposite backfat thickness phenotypes. The total number of paired-end reads produced for each sample was in the range of 39.29–49.36 millions. Approximately 188 genes were differentially expressed in adipose tissue and were enriched for metabolic processes, such as fatty acid biosynthesis, lipid synthesis, metabolism of fatty acids, etinol, caffeine and arachidonic acid and immunity. Additionally, many genetic variations were detected between the two groups through pooled whole-genome resequencing. Integration of transcriptome and whole-genome resequencing data revealed important genomic variations among the differentially expressed genes for fat deposition, for example, the lipogenic genes. Further studies are required to investigate the roles of candidate genes in fat deposition to improve pig breeding programs.

## Introduction

Pigs are an important source of meat worldwide [1]. The fatness traits of pigs impact growth rate, meat quality and reproductive performance [2].The thickness of backfat is a good indicator for fat deposition as it correlates well with over all body fat, carcass cross-sectional fat area ratios and intramuscular fat content[3]. Elucidating the role of specific genes in lipid metabolism and obesity is critically important and feasible to achieve in pigs. The similarity of pigs to humans in terms of body size and other physiological/anatomical features, including their innate tendency to over-consume food, has made pigs an important animal model for the study of the genetic basis of metabolic diseases such as obesity, type II diabetes, metabolic syndrome and atherosclerosis[4].

Adipose tissues, liver and skeletal muscles are the most important organs in animals with respect to whole body lipid metabolism [1,5]. Lipid is stored in adipose tissue to provide energy. Meanwhile the adipose tissue is the main location for *de novo* fatty acid synthesis, and is the major source of circulating free fatty acids (FFAs) in pigs[6]. Lipid in adipose tissue can be hydrolyzed to glycerol and FFAs. FFAs, combined with plasma albumin, can be transported and used as an energy source through oxidation [7]. Furthermore, it also produces adipocytokines, peptide hormones and resistin, and hence functions as an endocrine organ[8].

Technological advances now allow sequencing to be performed more economically and efficiently than ever before, providing excellent opportunities to investigate biological problems. The transcriptome of pig adipose tissue has recently been studied using RNA sequencing (RNA-seq). Differences in adipose tissue transcriptomes from different breeds and different growth phases were studied[9]. Functional differences were reported for the different types of adipose tissue[10],and adipose tissues in two phenotypically different individuals were compared for different fat deposition and fatty acid composition in muscle [11,12]. Meanwhile, whole genome resequencing has also been employed in livestock species such as cattle[13] and chicken[14].

Previous RNA-Seq studies to analyze the regulation of adipose deposition in pigs have either used only a very small number of adipose tissue samples or have lacked adequate control of genetic backgrounds. In our study, we applied RNA-Seq to obtain adipose tissue transcriptomes from two pair full-sibs and from one pair of unrelated pigs, the pigs in each pair having opposite backfat thickness phenotypes. We also used whole-genome resequencing technology to investigate the genetic basis for variations in expression among the differentially expressed genes.

## Materials and Methods

### Experimental design, animals and phenotypes

A Songliao black pig resource population (around age, 216 days which ranged from 211 to 218 days and average live weight, 103.9 kg which ranged 85.0 kg to 105.4 kg) was used in this study. All animals were housed in consistent and standard environmental conditions, given the same diet and *ad libitum*. Pedigree information was available for all animals. Live backfat thickness of 53 individuals was measured on the last 3^rd^ and 4^th^ ribs using the B mode real time ultrasound (HS1500, Honda, Japan) to choose pairs with divergent backfat phenotypes. Selection methods and standards were the same as in our previous study [15]. Based on our criteria, three pairs of pigs, two of which were full-sibs, were chosen with each pair having opposite backfat thickness phenotypes. All chosen pigs were stunned with a captive bolt, exsanguinated and slaughtered in commercial abattoir called Beijing Huadu Sunshine Food co., LTD. Slaughter house management gave the necessary permissions for slaughter. The chosen pigs were slaughtered according to guidelines of operating procedures of pig-slaughtering (GB/T 17236–2008), which was promulgated by General Administration of Quality Supervision, Inspection and Quarantine of the People's Republic of China (AQSIQ) and Standardization Administration of the People’s Republic of China(SAC).All efforts to minimize animal suffering were made during the study. The whole procedure for collection of the tissue samples of all animals was by our researchers. This study was approved specifically by the Animal Welfare Committee of China Agricultural University (Permit number: DK996). Adipose samples from backfat between the last 3^rd^ and 4^th^ ribs were frozen in liquid nitrogen immediately after slaughter and stored at -80°C until used for RNA extraction. Liver samples were collected and stored at -20°C until used for DNA extraction.

**Fig 1:**
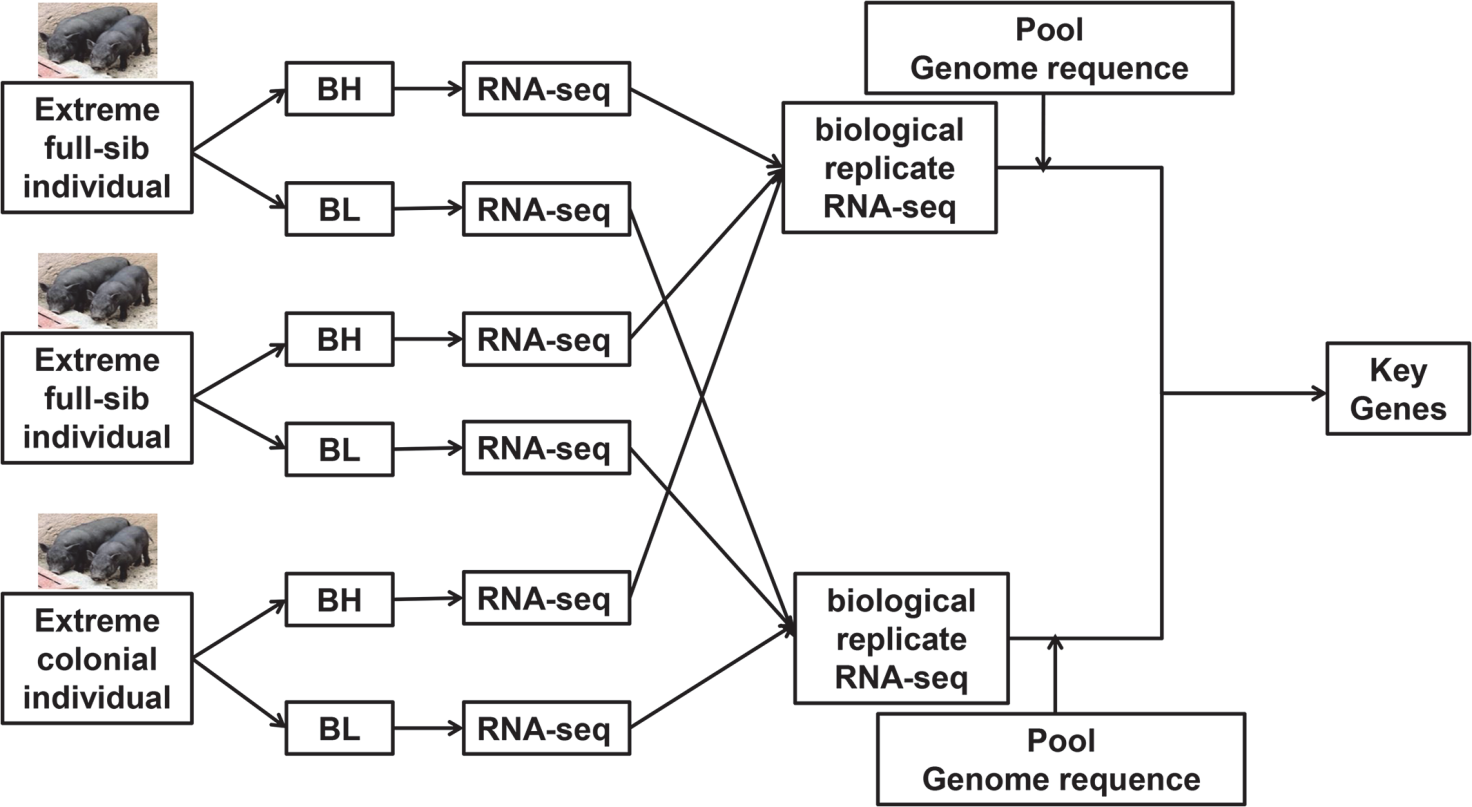
Experimental study design. Schematic picture of the experimental design. Two of three pairs extreme individuals are full-sibs. BH: Songliao Black pigs with higher backfat of live group. BL:Songliao Black pigs with lower backfat of live group.

Our experimental design is shown in Fig 1. We identified differentially expressed genes (DEGs) between Songliao Black pigs with higher backfat of live (BH) and BL Songliao Black pigs with lower backfat of live (BL) groups. We also used whole-genome resequencing to identify mutations in the two pig groups to identify and annotate genetic variation influencing fat deposition. We integrated transcriptome and genomic resequencing data to identify key genes and mutations affecting swine fat deposition.

### mRNA Library construction and sequencing

One paired-end mRNA library was constructed from the adipose tissues of each of the six pigs. Purified mRNA was first fragmented using an RNA fragmentation kit (Ambion, Austin, TX, USA). Paired-end libraries were prepared following the Illumina paired-end library preparation protocol (Illumina, San Diego, California, USA). The libraries were sequenced using a multiplexed paired-ends protocol with 180bp of data collected per run on an Illumina HiSeq 2000 (Illumina). The average insert size for the paired-end libraries was 180 bp.

### DNA library construction and sequencing

Sample preparation, cluster generation and sequencing were performed according to Illumina protocols. Total DNA from each pig was quantified, evenly mixed together for each group (BH and BL) and used for whole-genome resequencing. Purity and yield were checked using a 2100 Bioanalyzer (Agilent Technologies, Santa Clara, California, USA). Briefly, two multiplexed paired-end libraries were prepared and sequenced using a HiSeq2000 (Illumina). Average insert size for each library was 220 bp.

### Bioinformatic analysis

The raw data were subjected todata quality evaluation, filtering and trimming before mapping on the swine reference genome. After the data quality control process, we used TopHat software for mapping short-reads and easyRNASeq software to quantify the raw reads. Differentially expressed genes (DEGs) were discovered using NOISeq [16]. NOISeq is a nonparametric approach for the identification of differentially expressed genes. Because of the noise distribution from the actual data, the NOISeq method could better adapt to the size of data set and was more effective in controlling the rate of false discoveries. A threshold of 0.8 was used for this probability, meaning that the gene is four times more likely to be differentially expressed than non-differentially expressed, as an adjusted P-value threshold of 0.001 for the other methods[17]. Functional enrichment analysis of the DEGs were performed in the DAVID system [18] and functional protein interaction networks were constructed in the STRING database[19]. Details of the RNA-Seq analysis are in our previous publication[15]. BWA software was used to map genomic resequencing data to the reference pig genome (Sscrofa10.2) with default parameters[20]. Quality score recalibration, local indel realignment and removal of duplicates from alignments was performed using GATK[21]. Single nucleotide variants (SNVs) and indel variants were called using GATK.

### Quantitative PCR analysis

To validate changes in transcript levels between BH and BL groups (n = 3)(for genes with functions related to fatty acid synthesis), six genes, including *ACACA*, *LDHA*, *ELOVL6*, *CYP1A2*, *PDK1* and *SCD*, were selected and quantified using qRT-PCR. Total RNA was extracted from adipose tissue and converted to cDNA using a Revert Aid First Strand cDNA Synthesis Kit (Thermo Fisher Scientific, Waltham, MA, USA) following the manufacturer’s protocol. The cDNA samples were then analyzed by RT-PCR using a Light Cycler 480 Real-Time PCR System (Roche, Hercules, CA, USA). The RT-PCR reactions were performed in a final volume of 20 μl using the Roche SYBR Green PCR Kit (Roche, Hercules, CA, USA) according to the manufacturer’s instructions. The RT-PCR primers designed for the six genes are listed in S1 Table. Gene expression values for qRT-PCR were normalized using the housekeeping gene, *GAPDH*. Triplicate qRT-PCRs were performed on each cDNA and the average Ct was used for further analysis. The relative quantification values were calculated using the 2^-ΔΔCt^ method.

## Results

### Analysis of RNA Deep Sequencing Data

**Fig 2:**
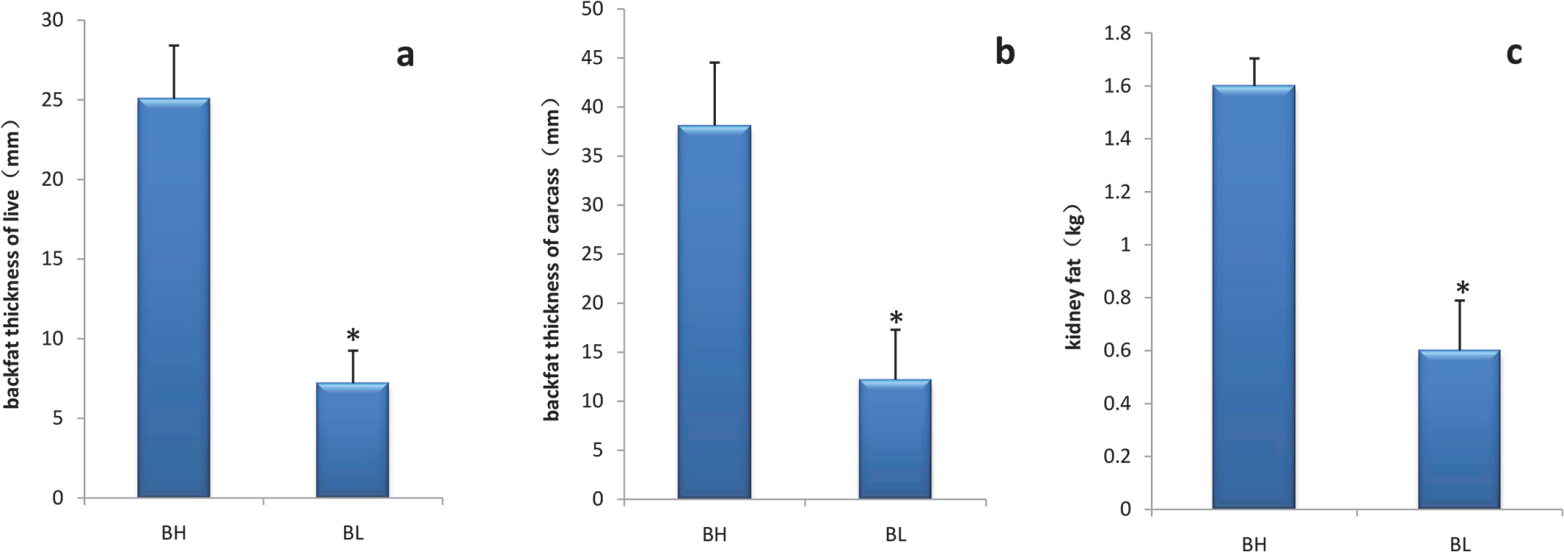
The traits of backfat thickness and kidney fat in BH and BL. The three traits are backfat thickness of live animals (a), backfat thickness of carcass (b) and weight of kidney fat (c). BH and BL are pigs with higher/lower thickness backfat of live using B-Ultrasound respectively. *: significantly different (p < 0.05)

Similar to our previous study, live individuals in the BH group had twice the backfat thickness compared with that in the BL group[15].Other traits related to fat deposition, such as carcass backfat thickness and kidney fat weight, were also divergent in the BH and BL groups (Fig 2). Sequencing six samples yielded average 43.9 millions 90 bp paired-end reads and an around 80% of reads were mapped against the reference pig genome assembly, Sscrofa10.2, using TopHat software. 65.20%–72.90% of total tags were aligned to the CDS region of the reference pig genome. The ratio of reads mapped in the 3′-UTR, intron and 5′-UTR regions was diminished (Table 1).

**Table 1:** Summary of RNA-seq alignment.

Sample ID	Total tags (Million)	Mapping rate%	CDS exons (%)	5' UTR (%)	3'UTR (%)	Intron (%)
**H611**	44.92	79.98	65.20	1.73	11.90	5.42
**H1103**	42.73	80.91	67.88	1.76	11.74	4.70
**H710**	47.36	82.69	72.90	1.57	10.20	3.51
**H712**	43.69	85.79	71.00	1.08	10.32	4.52
**H906**	45.26	82.96	68.69	1.54	11.47	4.50
**H909**	39.29	85.79	66.94	1.05	11.20	6.04

BH: Songliao Black pigs with higher backfat of live group

BL:Songliao Black pigs with lower backfat of live group

H710, H712 and H906, H909 are two pair full-sibs pigs. H710, H906,H611 and H712, H909, H1103 are the BH and BL groups respectively.

Use the Ensembl V68 as the reference genome annotation to classify the mapping tags into the different regions. Ratio of the tags mapping on the subregion of the gene was calculated as the tags on each region divided by the total tags on the whole genome.

To normalize gene expression across different samples, the method of Trimmed Mean of M-values (TMM) was used to quantify transcript expression levels. The total number of expressed genes in different animals was similar and ranged from 17,175 to 17,715 (S2 Table). Correlations between biological replicate samples had very high reproducibility. The deep sequencing data of total RNA have been submitted to NCBI Sequence Read Archive (SRA) under Accession no.SRP035333, Bioproject: PRJNA234335.

To validate the RNA-seq results, on the basis of differential expression, a total of six genes with functions related to fatty acid synthesis, including *ACACA*, *LDHA*, *ELOVL6*, *CYP1A2*, *PDK1* and *SCD*, were selected and their expression assayed byRT-PCR. The commonly used reference gene, *GAPDH*, was used for validation. For all six genes, the RT-PCR fold-change ratios between BH and BL groups were consistent with the RNA-Seq data (S1 Fig).

### Differential gene expression and biological function analysis

**Fig 3:**
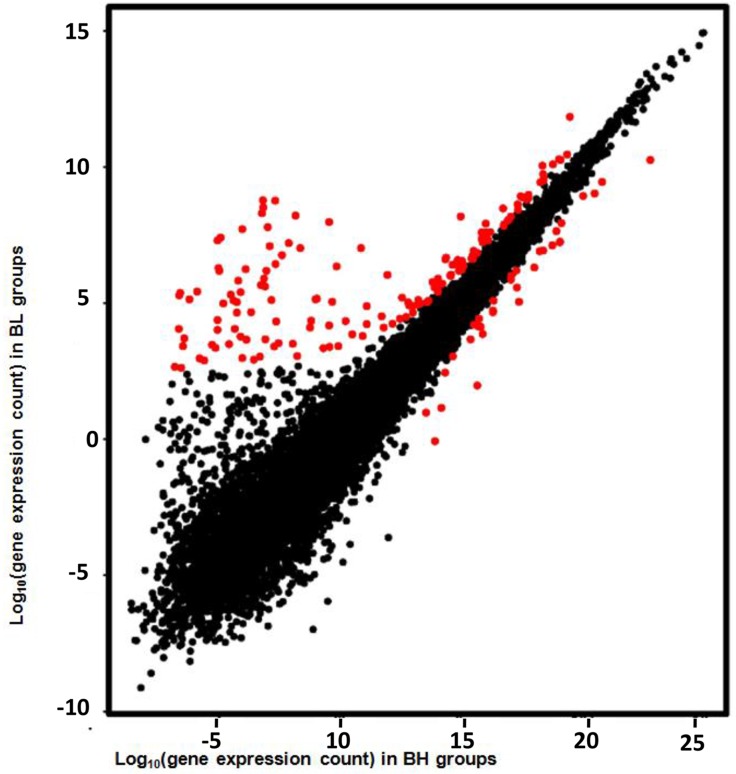
Gene expression level in BH and BL groups. A. X-axis plots gene expression counts in the BH group after TMM quantification and the y-axis plots gene expression counts in the BL group after TMM quantification. The red points indicate significantly differentially expressed genes.

**Fig 4:**
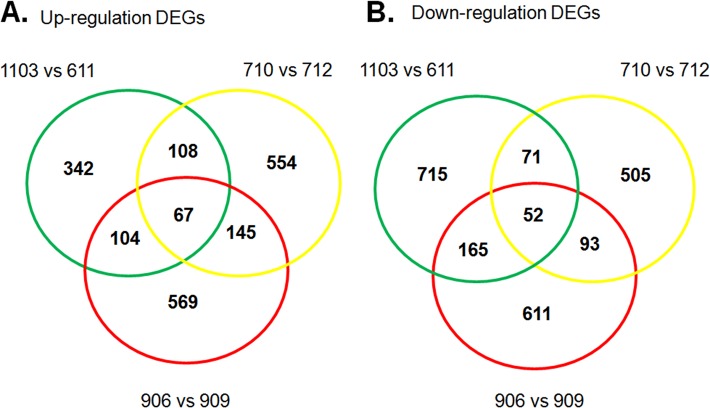
Venn diagrams showing differentially expressed genes (DEGs) among each of three pairs. A. Up-regulated genes in BH compared with BL in three pairs. B. Down-regulated genes in BH compared with BL in three pairs.

In our analysis, we treated the high/low backfat thickness samples as the biological samples as they showed similar phenotypes in each group (BH or BL). A total of 188 DEGs, including 32 up-regulated and 156 down-regulated genes, were selected (Fig 3). These DEGs were catalogued according expression in BH and BL adipose tissue (S3 Table). Moreover, we also analyzed the DEGs between pigs in each pair and then identified the DEGs that were common among the three different pairs (Fig 4). The DEG details, including gene name, expression level and statistical information, were shown in the S4 Table. Because of the sample limitations of the DEGs for each pair, we only show DEGs and their functional analysis that were obtained by treating three pairs as the three biological replicates.

**Fig 5:**
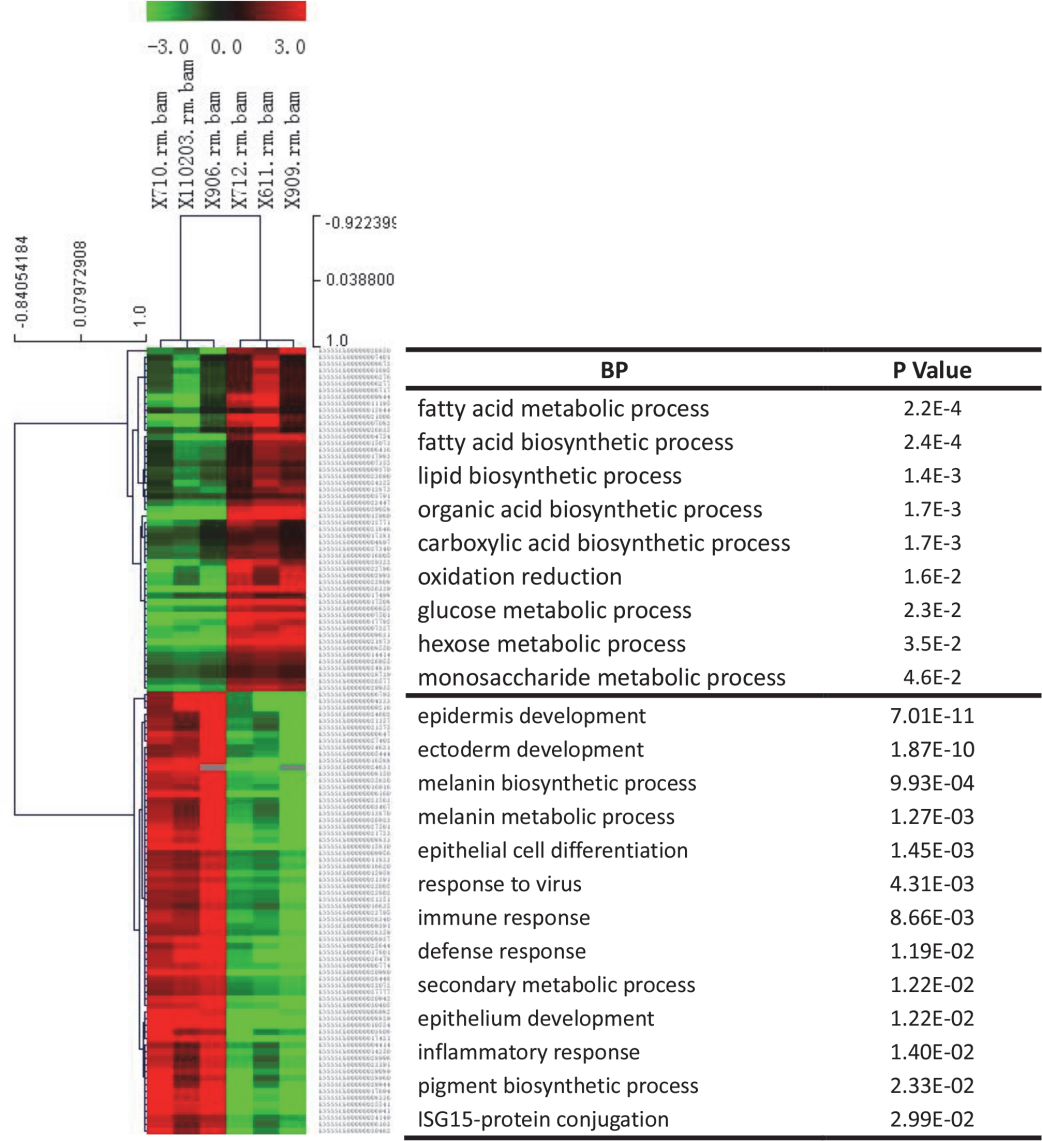
Heatmap of differentially expressed genes (DEGs) in adipose tissue and GO analysis. The heatmap was generated by hierarchical cluster analysis of DEGs. The processes listed opposite the upper half of the heatmap are gene ontology annotation processes of the up-regulated genes [biological process (BP)]. The lower processes are those (BP) of the down-regulated genes.

About 27 of 28 highly expressed genes in the BH group were mapped to GO and KEGG pathways in DAVID. The functional enrichment of pathways (Table 2) and biological functions (Fig 5) included fatty acid biosynthesis, the insulin signaling pathway, biosynthesis of unsaturated fatty acids, lipid synthesis, fatty acid metabolic process, response to hormone, lipase activity, and saccharide metabolic process. These results showed that the enzymes involved in fatty acid and unsaturated fatty acid synthesis, such as *ACACA*, *FASN*, *SCD* and *ELOVL6*, were highly expressed in the BH group. Meanwhile, several genes in the insulin signaling pathway were significantly increased in the BL group. From 122 down regulated genes, 119 were assigned to gene ontology categories, such as molecular metabolism, receptor signaling pathway, immune response and lipid metabolism. Biological processes (Fig 5) and pathways (Table 2) relating to the metabolism of several compounds, including retinol, caffeine, arachidonic acid, and drugs were enriched in cytochrome P450 (CYP) family genes. The details of gene ontology categories were provided in the S5 Table.

**Table 2:** Pathways (P <0.05) enriched in differentially expressed genes (DEGs) in adipose tissue(BH vs. BL).

	pathway	Gene	P value
**Up-regulated**	Fatty acid biosynthesis	ACACA 、FASN	1.6E-2
**Down-regulated**	Vascular smooth muscle contraction	ACTA2 、ACTG2、CYP4A11、CYP4A22、MYL9、 Ppp1r14a、RAMP1	9.6E-5
	Retinol metabolism	Cyp2A13、CYP2A7、 CYP2B6、CYP4A11、CYP4A22	4.7E-4
	PPAR signaling pathway	CYP27A1、CYP4A11、CYP4A22、PCK1	1.1E-2
	RIG-I-like receptor signaling pathway	ISG15、CXCL10、 IRF7、NFKBIA	1.2E-2
	Toll-like receptor signaling pathway	CXCL10、 IRF7、NFKBIA、CXCL9	3.1E-2
	Caffeine metabolism	CYP2A13、CYP2A7	4.7E-2

BH: Songliao Black pigs with higher backfat of live group

BL:Songliao Black pigs with lower backfat of live group

**Fig 6:**
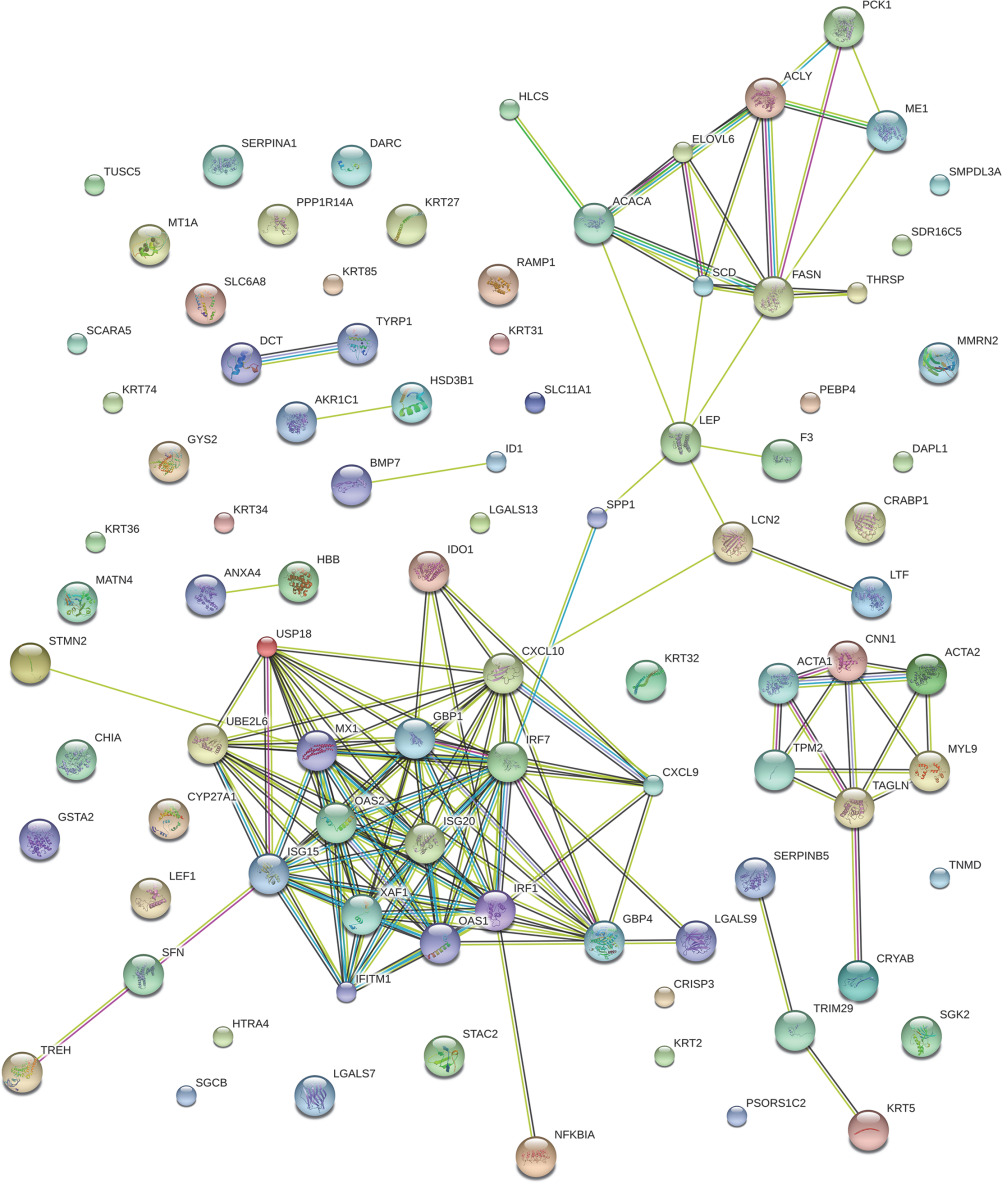
STRING analysis shows that DEGs are involved in known and predicted protein-protein interactions. STRING is used to analyze of DEGs in adipose tissue between BH and BL. The network nodes stand for those genes shown in S3 Table. Lines of different colors represent seven types of evidence used in predicting associations. Red line: fusion evidence; green line: neighborhood evidence; blue line: co-occurrence evidence; purple line: experimental evidence; yellow line: text mining evidence; light blue line: database evidence and black line: co-expression evidence.

STRING protein interaction network analysis generated a network from the genes that were up-regulated in BH compared with BL that were related to *de novo* fatty acid synthesis, from providing the substrate to participating in synthesis. Another network is related to immune action through three systems: 1) direct involvement in the antiviral process, 2) regulation of interfere on antiviral effectors, 3) functions in ubiquitination by immune and proinflammatory responses (Fig 6). Most genes in the immune action network are down-regulated in the BH group compared with the BL group.

### Genome sequencing, SNP/indel detection, and functional annotation of genomic variation

**Fig 7:**
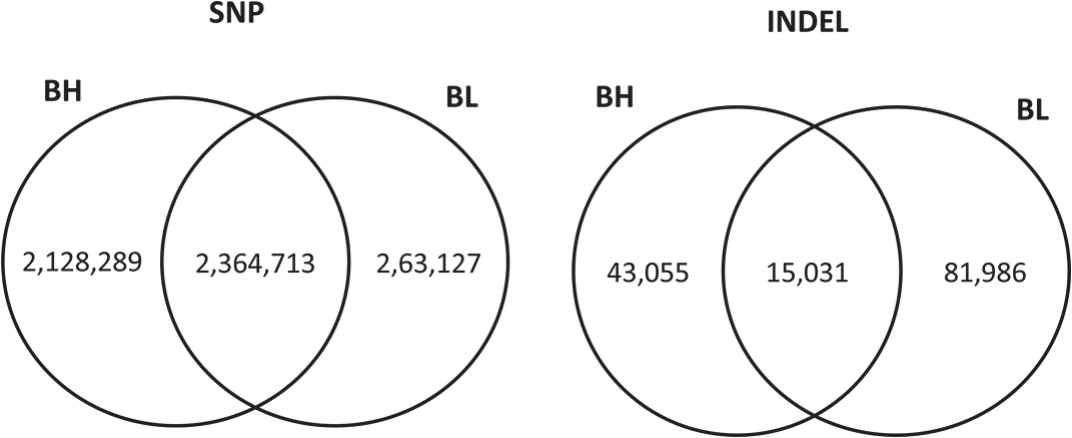
The venn diagram of SNPs and Indels in BH and BL. The number of SNPs and Indels in genome located in BH and BL groups.

Each whole genomic resequencing pooled sample were harvested to about 25G clean data with average 10×for each pool. Complete data sets of whole genomic resequencing have been submitted to NCBI SRA under Accession no. SRP040044, Bioproject: PRJNA240950. Using GATK, we identified single nucleotide polymorphisms (SNPs) and indels in BH and BL groups, some of which were common (Fig 7). The number of homozygous SNPs in BH and BL groups, was smaller than that of heterozygous SNPs, with a ratio of 1:1.46 and 1:1.36 (homozygous: heterozygous), respectively, but among the indels, the result was the opposite (2.63:1 and 2.61:1). Most of the SNPs and indels are located between genes or within intergenic regions and introns.

Because the two groups have opposite backfat thickness phenotypes, we focused on the SNPs and indels that were different between groups. The SNPs that were only in BH or BL were annotated to 21,134 and 21,623 genes, respectively, using the NCBI Reference sequence database (S6 Table). Non-synonymous (NS) SNPs and indels within a coding DNA sequence (cIndel) may affect gene expression or function. We detected 5,099 NS SNPs in 2753 genes and 145 cIndels in 104 genes in the BH group. Similarly, we detected 6,010 NS SNPs in 3116 genes and 223 cIndels in 139 genes in the BL group. In total, there were 2818 and 3198 genes with NS SNPs/cIndels in the BH and BL groups, respectively. After conversion to homologous human genes, we conducted a DAVID functional annotation clustering analysis of genes containing variants (NS SNPs/cIndels) to identify molecular functions (MF), biological processes (BP), cellular components (CC) and KEGG pathways (S7 Table). The genes with variants (NS SNPs/cIndels) were found in numerous pathways, including synthesis, metabolism, oxidation reduction, enzymes activity, molecule transport and binding. The most significant pathway was olfactory transduction in both BH and BL, and is involved with 107 and 110 genes in BH and BL, respectively. Sixty and 67 genes were detected in the lipid related process such as lipid biosynthesis, lipid transport, lipid localization, lipid binding, fatty acid metabolic process, and regulation of lipid kinase activity. NS SNPs/cIndels genes were significantly enriched in the linoleic acid metabolism, fatty acid metabolism and starch and sucrose metabolism which are very important in the lipid metabolism (S7 Table).

### Mutations in DEGs supported by genomic resequencing

A large number of SNPs and indels were detected in adipose tissue DEGs in the BH and BL groups (S8 Table). The genetic variations included frame shift, splice site, UTR and non-synonymous coding lesions that may affect gene expression, function and even phenotype. As mentioned above, through DEG functional enrichment analysis, several genes regulating lipid processes were found. For these genes, genetic variation between BH and BL is particularly important. Almost all indels were found in introns except for two frame-shifts: one in the *ACACA* gene and one in the *CYS2* 3′-UTR. It is noteworthy that a great number of indels were identified in *ACACA* and *ELOVL6* genes. Many SNPs were detected in important regions of the key DEGs. For instance, *SCD*, a key gene in the biosynthesis of unsaturated fatty acids, had seven SNPs in the 3′-UTR. For *FASN*, a key gene in the biosynthesis of fatty acids, the genetic variation was more complex and included three 5′-UTR and nine non-synonymous coding SNPs. SNPs were similarly identified in other genes, such as *CYS2*, *LGALS13*, *ME1*, *ACLY* and *CYP2B22*. Genetic variations in introns and in the 5kb upstream and downstream regions of key genes were also abundant. We also matched our DEGs’ position to the QTLs related to fat traits in the pig QTLdb (http://www.animalgenome.org/cgi-bin/QTLdb/SS/index). There are overlapping regions in 13 DEGs and QTLs related to fat traits (S9 Table), including the important gene *ELOVL6*. Those mutations in both DEGs and QTL region related to fat deposition would be studied in the next step work.

## Discussion

Lipid metabolism is one of the hot research topics. We compared with several previous transcriptome studies [11,22–24] to narrow down candidate genes which are critical important for lipid metabolism. Based on these comparisons, we found 53 genes showed the consistent expression patterns across different pig breeds (S10 Table).

### Fatty acid synthesis

**Fig 8:**
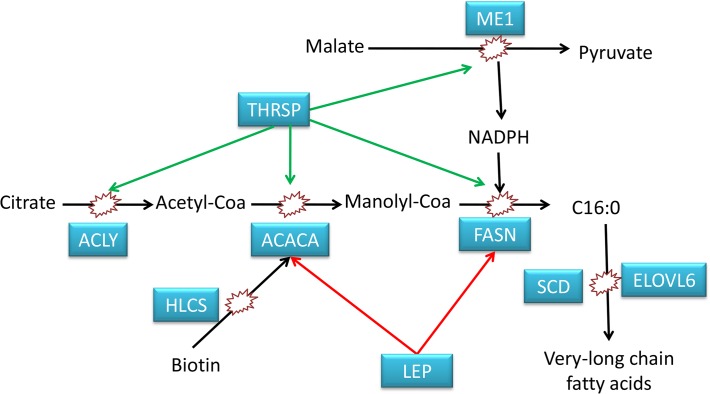
The function of DEGs in the de novo fatty acids synthesis. Black arrow means enzymes take part in the de novo fatty acids synthesis; red arrow means inhibiting effect; green arrow means promoting effect; blue quadrangle means up-regulation expression genes between BH and BL in adipose tissue

Previous studies have shown that almost all fatty acid synthesis occurs in adipose tissue [6]. The lipid synthetases, including *ACACA*, *FASN*, *SCD* and *ELOVL6* are up-regulated in BH. The *ACACA* gene encodes acetyl-CoA carboxylase alpha, which catalyzes the carboxylation of acetyl-CoA to malonyl-CoA. The acetyl-CoA carboxylase catalyzed reaction is the rate-limiting step and the key regulator of *de novo* fatty acid synthesis [25]. In past reports, the regulation of the *ACACA* transcript level depends on tissue, breed, and phenotype (fat versus lean)[25]. Consistent with our results, a significant up-regulation of the *ACACA* gene and of other genes involved in *de novo* lipogenesis was observed in muscle samples from fatter Duroc pigs in comparison to leaner ones[26]. FASN is also rate-limiting in *de novo* fatty acid synthesis as its main function is to catalyze the synthesis of palmitate from acetyl-CoA and malonyl-CoA[27]. Palmitic acid is normally the end point of *de novo* fatty acids biogenesis. *SCD* and *ELOVL6* are very important enzymes involved in fatty acid desaturation and elongation[28]. *SCD* is the rate-limiting enzyme in the biosynthesis of monounsaturated fatty acids [29]. In human, a high *SCD* activity is associated with a metabolic state favoring hepatic triglyceride accumulation and expansion of adipose triglyceride stores[30]. These genes are enriched in the biosynthesis of fatty acids and unsaturated fatty acids. *ME1* takes part in the tricarboxylic acid cycle to supply *NADPH* and to transport acetyl-CoA from mitochondria to the cytosol for the biosynthesis of fatty acids[31]. *ME1*, whose functions in glucose metabolism contribute to the initial steps of lipogenesis, is worth noting[32]. Consistent to previous study[11], *ME1* mRNA was more abundant in the fatty compared with the leaner group, which is consistent with the biological function of this gene; promoting adipose deposition[33,34].*ACLY* is the primary enzyme responsible in many tissues for the synthesis of cytosolic acetyl-CoA from citrate and CoA [35]. Leptin is a fat-derived cytokine whose levels directly correlate with fat mass and communicate the energy status of the organism to the brain[36]and inhibit *ACACA* and/or *FASN*[37]. In our study, mRNA level of Leptin is more abundant in BH group, similarity a full-sib study [11]. This negative feedback regulates body weight through inhibition of feed intake, and increasing fatty acid and glucose oxidation[38]. Leptin also increases glucose use, and this is consistent with the function of the insulin signaling pathway which is enriched with up-regulated genes expressed in adipose tissue. The insulin signaling pathway is an important regulator of lipid metabolism through increasing glucose uptake in fat, promoting the storage of substrates in fat by stimulating lipogenesis, and inhibiting lipolysis [39]. *THRSP* is abundantly expressed in lipogenic tissues and plays an important role in the biosynthesis of triglycerides with a medium-length fatty acid chain and in modulating lipogenesis[40]. In mammals, biotin serves as a covalently bound coenzyme for acetyl-CoA carboxylases (*ACACA*) and participates directly in fatty acid biosynthesis[41]. Holocarboxylase synthetase (*HLCS*) catalyzes the binding of biotin to acetyl-CoA carboxylases (*ACACA*) and, therefore, plays a pivotal role in biotin-dependent metabolic pathways[42]. The above mentioned genes, which are highly expressed in adipose tissue of BH compared with BL animals, take part in the *de novo* synthesis of fatty acid in three aspects: 1) lipid synthetase, 2)supply or transport of substrate, 3)direct or indirect regulatory function (Fig 8). Fatty acid synthesis in adipose tissue may, therefore, decide the backfat thickness. Much effort has been expended to find the effect of polymorphisms in the above genes on gene expression and fat-related traits in animals. Polymorphisms in the *ELOVL6* gene are associated with *ELOVL6* expression levels and fatty acid synthesis in pigs[43]. *ELOVL6* is up-regulated in BH pigs and we identified indels and SNPs within the gene. For *ACACA*, two SNPs have been associated with fatness and performance traits in Polish breeds and breed-specific differences in the transcript level were observed (fat versus lean) [25].Furthermore, we identified SNPs and indels in *ACACA*, which is up-regulated in our results. Five SNPs are located in the 3′-UTR of *ME1* and are associated with the *ME1* mRNA levels in muscular and adipose tissue and backfat thickness[44]. In our study, one *ME1*3′-UTR SNP was found between BH and BL groups and the expression level of *ME1* was different between BH and BL. This indicates that gene variation in key DEGs is very important. Because our groups are from a local Chinese breed of pig, the gene variation found in previous studies was not found in our study. Abundant genetic variations found in whole-genome resequencing provide a platform for more research. For fat deposition, synthesis of lipid is one key factor; lipid mobilization and fatty acid oxidation is another important area.

### Metabolism in adipose tissue

The adipocyte is not a passive lipid storage depot but a dynamic cell that plays a fundamental role in energy balance and overall body homeostasis[45]. The fat cell functions as a sensor of lipid levels, transmitting information to a neural circuit affecting hunger and satiety [46]. The PPAR signaling pathways regulate energy balance, lipid metabolism, cellular differentiation, and proliferation through PPARs, as members of the nuclear receptor superfamily[47,48].Activation of the PPAR signaling pathway promotes fatty acid oxidation[49].Genes in the PPAR signaling pathway and genes involved in fatty acid metabolism are down-regulated in adipose tissue between high and low backfat animals in cattle[50]. *CYP2A13*, in the PPAR signaling pathway, is down-regulated in adipose tissues between Korean native (fat) and Yorkshire (lean) pigs[51]. Consistent with our results, the PPAR signaling pathway and *CYP2A13* are down-regulated in adipose tissue between BH and BL. This indicates a higher fatty acid metabolism in adipose tissue in the BL group. Retinoids (vitamin A) are crucial for most forms of life[52], and many studies have identified connections between retinoid and lipid metabolism[53]. Retinoids regulate metabolism by activating specific nuclear receptors, including the retinoic acid receptor (RAR) and the retinoid Xreceptor (RXR), an obligate heterodimeric partner for other nuclear receptors, including PPARs, and help to coordinate energy balance[54].Retinoid metabolism was down-regulated in adipose tissue and up-regulated in liver (BH vs. BL) and may cause the difference in lipid metabolism between the two groups. Meanwhile, the P450 gene family is activated by RXR and PPARs[55]. The P450 genes play critical roles in catalyzing reactions in the metabolism of drugs, environmental pollutants and other xenobiotics and in the oxidation of unsaturated fatty acids to intracellular messengers[56]. Several P450 genes were found at different expression levels between Korean native (fat) and Yorkshire (lean) pigs[51]. In our results, some P450 genes behave similarly.

### Immune response and metabolic regulation in adipose tissue

The immune response and metabolic regulation are highly integrated. Adipose tissue is not an immune organ, but provides a close relationship between the immune system and metabolism[57].As above mentioned, the DEGs and pathways identified in this study regulate metabolism, especially fatty acid metabolism. Genes related to the immune system, such as *NFKBIA*, *IRF1*, *MX1* and *OAS1* show a significantly higher level of expression in the adipose tissue of BL pigs (the leaner group). Adipocytokines are a key component not only of metabolic regulation, but also of the immune response. Leptin, an abundant adipocyte product, represents one of the best examples of an adipocytokine. Leptin is involved in the control of energy expenditure, lipid and carbohydrate metabolism, which are mentioned above. Leptin also regulates endocrine and immune functions. Genetic defects of leptin or leptin receptors leads to reduced macrophage phagocytosis and the expression of proinflammatory cytokines, both *in vivo* and *in vitro*, and exogenous leptin up-regulates these[58]. These results indicate that leptin is up-regulated in inflammatory immune responses. But in our results, the mRNA level of leptin and of immune response-related genes was the opposite. Another way that metabolic and immune processes are regulated by lipids involves transcription factors, like PPARs and LXRs. Ligands to all three family members suppress *NFKB*, and then induce the production of proinflammatory cytokines. But unliganded *PPARβ* has an inflammatory function. In our results, both the PPAR signing pathway and *NFKBIA* are down-regulated in adipose tissue[59]. Studies demonstrate that changing the fatty acid composition of immune cells affects phagocytosis and other functions[60]. Candidate genes affecting fatty acid composition, such as *SCD* and *ELOVL6*, show different mRNA levels in adipose tissues between BH and BL. This is another example of the immune response and metabolism having countless ties. In our RNA-seq results, DEGs in adipose tissue included genes related to the immune response and metabolism. The specific interactions among them are not clear, but these data provide candidates for future studies.

## Conclusions

Here we showed whole genome expression differences in adipose tissues and genetic variation among pigs with opposite backfat thickness phenotypes. RNA-seq provided a high resolution map of transcriptional activities and around 80% of the total reads could be mapped to annotated references. Animals are classified into two groups according to their backfat thickness and 188 genes were found to be differentially expressed between groups. The identification of numerous DEGs confirms the key pathways and gene networks related to lipid synthesis and metabolism. The DEGs such *ACACA*, *FASN*, *SCD* and *ELOVL6* in *de novo* fatty acid synthesis in adipose tissue may affect backfat thickness. We also sequenced the whole genome of BH and BL mix pools. By comparison with the reference genome sequence, we also identified 4,493,002 SNPs and 68,276 indels in BH and 5,027,840 SNPs and 97,017 indels respectively. Genetic variation of this study, especially in DEGs, will provide valuable information for function studies, as well as for marker development associated with traits related to lipid deposition. Further studies are required to investigate the roles of candidate genes in fat deposition to improve pig breeding programs.

## Supporting Information

S1 Fig Comparison of qPCR and RNA-Seq expression ratios (BH and BL groups) for the selected genes. (TIF)

S1 Table qRT-PCR primers for six genes related to lipid metabolism. (XLS)

S2 Table Gene expression count in the six samples. (XLS)

S3 Table DEGs between BH and BL in adipose tissue. (XLS)

S4 Table DEGs common among the three different pairs. (XLSX)

S5 Table Details of gene ontology categories for differently expression genes. (XLSX)

S6 Table SNPs and indels only in BH and BL annotated in genes. (XLS)

S7 Table Genes with NS or cIndel annotated in DAVID. (XLSX)

S8 Table SNP and indels by type in differently expression genes. (XLSX)

S9 Table Information of overlapping between DEGs and QTLs related to fat traits. (DOCX)

S10 Table Detail information of common DEGs compared with other studies. (DOCX)
