# Supplementary figures and images for: Integration of Transcriptome and Whole Genomic Resequencing Data to Identify Key Genes Affecting Swine Fat Deposition
Source: PLoS One. 2015 Apr 7;10(4):e0122396. doi: 10.1371/journal.pone.0122396 (PMC4388518; doi:10.1371/journal.pone.0122396)

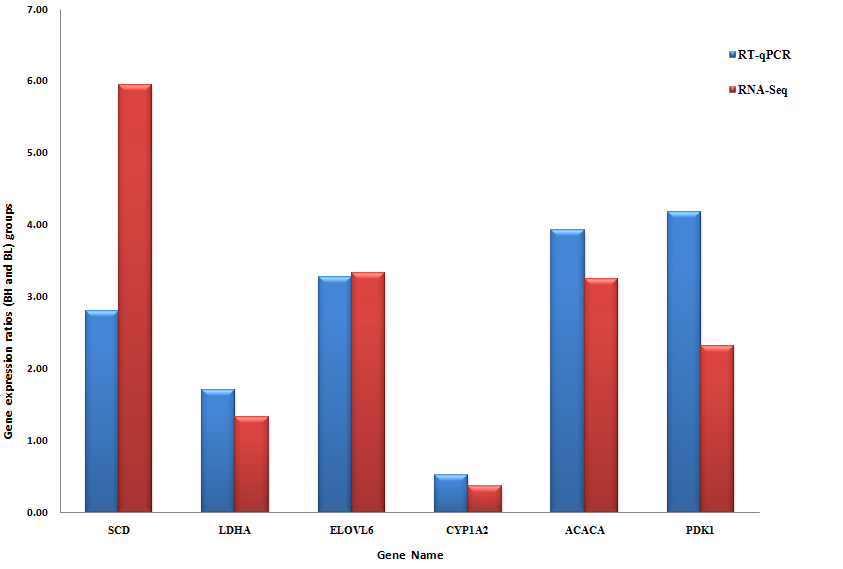

Supplement: S1 Fig — (TIF) [file pone.0122396.s001.tif]
