# Supplementary material for: Integration of Transcriptome and Whole Genomic Resequencing Data to Identify Key Genes Affecting Swine Fat Deposition
Source: PLoS One. 2015 Apr 7;10(4):e0122396. doi: 10.1371/journal.pone.0122396 (PMC4388518; doi:10.1371/journal.pone.0122396)
Supplement: S9 Table — (DOCX) [file pone.0122396.s010.docx]

| Gene ID | Gene symbol | Gene Position | QTL region | fat deposition traits of QTL effect |
| --- | --- | --- | --- | --- |
| ENSSSCG00000005638 | LCN2 | Chr1: 302600057-302605205 | Chr1：302600678-302605199 | Fat to meat ratio; Fat-cuts percentage; Lean meat percentage |
| ENSSSCG00000006793 | novel gene | Chr4: 119378021-119395675 | Chr4: 119378021-119395675 | Backfat at last lumbar; Backfat thickness (EBV); backfat at last rib |
| ENSSSCG00000009216 | SPP1 | Chr8: 140273369-140315456 | Chr8: 135387386-140315515 | Loin muscle area |
| ENSSSCG00000025541 | ELOVL6 | Chr8：120119623-120219840 | Chr8：119964648-120553940 | Average backfat thickness |
| ENSSSCG00000026805 | novel gene | Chr15: [133116446-133132610](http://www.ensembl.org/Sus_scrofa/Location/View?db=core;g=ENSSSCG00000026805;r=15:133116446-133132610;t=ENSSSCT00000024321) | Chr15: 132895540-137368522 | Average glycolytic potential |
| ENSSSCG00000025058 | NRAMP1 | Chr15: [133452329-133456736](http://www.ensembl.org/Sus_scrofa/Location/View?db=core;g=ENSSSCG00000025058;r=15:133452329-133456736;t=ENSSSCT00000024413) | Chr15: 132895540-137368522 | Average glycolytic potential |
| ENSSSCG00000023379 | UBE2L6 | Chr2: 12987517-13002993 | Chr.2: 13002192-13002292 | Backfat weight; Percentage of backfat and leaf fat in carcass |
| ENSSSCG00000006277 | SPIDR | Chr4: 87368561-87610320 | Chr.4: 87410825-87410925 | backfat at last rib; Backfat at tenth rib; Intramuscular fat content |
| ENSSSCG00000002476 | SERPINA1 | Chr7: 122616093-122621528 | Chr.7: 122479997-122645103 | Intramuscular fat content; |
| ENSSSCG00000003006 | CYP2B22 | Chr6: 44929854-44978800 | Chr.6: 44884131-45072146 | Abdominal fat weight; External fat on ham; Shoulder external fat weight; Backfat weight; backfat above muscle dorsi; Backfat at tenth rib; Average backfat thickness |
| ENSSSCG00000012853 | IRF7 | Chr2: 299444-302179 | Chr2: 222025-476748 | Backfat thickness (EBV) |
| ENSSSCG00000029879 | LTF | Chr13: 32577739-32609442 | Chr.13:32409037-32745853 | Average backfat thickness |
| ENSSSCG00000005474 | SAL1 | Chr1: 284447110-284451960 | Chr.1: 284121929-284536735 | Abdominal fat weight; Backfat weight |
