# Supplementary material for: Integration of Transcriptome and Whole Genomic Resequencing Data to Identify Key Genes Affecting Swine Fat Deposition
Source: PLoS One. 2015 Apr 7;10(4):e0122396. doi: 10.1371/journal.pone.0122396 (PMC4388518; doi:10.1371/journal.pone.0122396)
Supplement: S10 Table — (DOCX) [file pone.0122396.s011.docx]

| **Compare study** | **Commom DEGs** | **Description** |
| --- | --- | --- |
| High fat deposition VS. Low fat deposition [[11](#_ENREF_2)] | PEBP4 | phosphatidylethanolamine-binding protein 4 |
|  | SAL1 | salivary lipocalin |
|  | TUSC5 | tumor suppressor candidate 5 |
|  | LEP | leptin |
|  | FASN | fatty acid synthase |
|  | ACACA | acetyl-CoA carboxylase alpha |
|  | TRIM29 | tripartite motif containing 29 |
|  | ID1 | inhibitor of DNA binding 1, dominant negative helix-loop-helix protein |
|  | SERPINA1 | serpin peptidase inhibitor, clade A |
|  | UBE2L6 | ubiquitin-conjugating enzyme E2L 6 |
|  | SPP1 | secreted phosphoprotein 1 |
|  | SDR16C5 | short chain dehydrogenase/reductase family 16C, member 5 |
|  | BMP7 | bone morphogenetic protein 7 |
|  | USP18 | ubiquitin specific peptidase 18 |
|  | MATN4 | matrilin 4 |
|  | DARC | Duffy blood group, chemokine receptor |
|  | IDO1 | 窗体顶端  indoleamine 2,3-dioxygenase 1 |
|  | SCARA5 | scavenger receptor class A, member 5 |
|  | MT-2B | metallothionein isoform |
|  | CYP27A1 | cytochrome P450, family 27, subfamily A, polypeptide 1 |
|  | LGALS9 | lectin, galactoside-binding, soluble, 9 |
|  | LTF | lactotransferrin |
|  | CYP2B22 | cytochrome P450 2B22 |
|  | LGALS13 | lectin, galactoside-binding, soluble, 13 |
|  | MX1 | myxovirus (influenza virus) resistance 1 |
|  | PSORS1C2 | psoriasis susceptibility 1 candidate 2 |
|  | ANXA4 | annexin A4 |
|  | STMN2 | stathmin-like 2 |
|  | ME1 | malic enzyme 1, NADP(+)-dependent, cytosolic |
| Jeju Native  Pig (obesity) VS. Berkshire (lean) [[22](#_ENREF_1)] | LOC100736878 | 窗体顶端  Ig kappa chain V-II region RPMI 6410-like |
|  | LOC100627004 | interferon-induced transmembrane protein 1-like |
|  | LOC100516758 | coronin-6-like |
|  | PCK1 | phosphoenolpyruvate carboxykinase 1 |
|  | LOC102161607 | immunoglobulin lambda-like polypeptide 5-like |
|  | SAL1 | salivary lipocalin |
|  | OTOR | otoraplin |
|  | TNMD | tenomodulin |
|  | MT1A | metallothionein 1A |
|  | LCN2 | lipocalin 2 |
|  | LOC100153946 | CCAAT/enhancer binding protein (C/EBP), delta |
| Korean native pig (fatty) VS. Yorkshire (lean) [2[3](#_ENREF_3)] | TPM2 | tropomyosin 2 |
|  | USP18 | ubiquitin specific peptidase 18 |
| Rongchang piglet VS. Landrace [[24](#_ENREF_4)] | OAS2 | 2'-5'-oligoadenylate synthetase 2, 69/71kDa |
|  | RSAD2 | radical S-adenosyl methionine domain-containing protein 2 |
|  | THRSP | thyroid hormone responsive |
|  | IRF1 | interferon regulatory factor 1 |
|  | HERC5 | HECT and RLD domain containing E3 ubiquitin protein ligase 5 |
|  | MT-2B | metallothionein isoform |
|  | OAS1 | 2'-5'-oligoadenylate synthetase 1, 40/46kDa |
|  | CYP2B22 | cytochrome P450 2B22 |
|  | LOC100621421 | acidic mammalian chitinase-like |
|  | CHAC1 | ChaC, cation transport regulator homolog 1 (E. coli) |
|  | CEBPD | 窗体顶端  CCAAT/enhancer binding protein (C/EBP) |
|  | XAF1 | XIAP associated factor 1 |
|  | TNMD | tenomodulin |
|  | LCN2 | lipocalin 2 |
|  | THRSP | thyroid hormone responsive |
|  | CHIA | chitinase, acidic |
|  | MX1 | myxovirus (influenza virus) resistance 1 |
|  | LGALS13 | lectin, galactoside-binding, soluble, 13 |
|  | SAL1 | salivary lipocalin |
|  | MMRN2 | multimerin 2 |
|  | MT1A | metallothionein 1A |
|  | USP18 | ubiquitin specific peptidase 18 |
